# Feeding strategy optimization in interaction with target seeding density of a fed-batch process for monoclonal antibody production

**DOI:** 10.1186/1753-6561-7-S6-P78

**Published:** 2013-12-04

**Authors:** Marie-Françoise Clincke, Grégory Mathy, Laura Gimenez, Guillaume Le Révérend, Boris Fessler, Jimmy Stofferis, Bassem Ben Yahia, Nicola Bonsu-Dartnall, Laetitia Malphettes

**Affiliations:** 1 Cell Culture Process Sciences Group, BioTech Sciences, UCB Pharma S.A., Braine L'Alleud, Belgium; 2 In-Process Analytics Group, BioTech Sciences, UCB Celltech, Slough, UK

## Background

Current trend towards Quality by Design (QbD) leads the process development exercise towards systematic experimentation, rational development, process understanding, characterization and control. In this study, an example of the application of QbD approach is given. Optimization of the feeding strategy and the target seeding density was performed and interactions of the two parameters were assessed in order to enhance cell growth and MAb productivity. The feeding strategy was optimized to take into account daily process performance attributes and associated nutrient needs of the culture to maintain a balance between metabolism and MAb productivity. For scale up the feed strategy was simplified to become independent of daily process performance attributes. Feed ranging studies were performed to assess the robustness of the process.

## Materials and methods

2L stirred tank bioreactors were run for 14 days in a fed-batch mode in a chemically defined medium. Feed was added daily from day 3 onwards. If required, antifoam C was added to the bioreactor by manual injections. DO, pH, and temperature were controlled at setpoint. DO was controlled using a multi-stage aeration cascade via a ring sparger. Viable cell concentration, cell viability, and average cell diameter were measured using a ViCell cell counter. The glucose, lactate, glutamine and ammonia concentrations were measured with a BioProfile Analyzer 400. On the day of harvest, the clarification was performed by centrifugation plus depth filtration. Monoclonal Antibody (MAb) concentration of the supernatant samples was quantified using Octet QK and Protein A high performance liquid chromatography.

## Results

### Interaction study between feeding strategy and Target Seeding Density (TSD)

**Figure 1:**
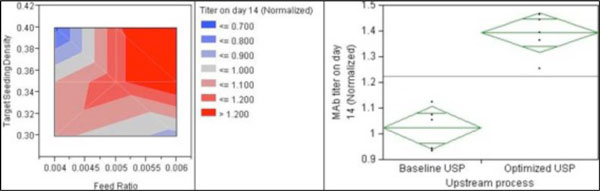
Impact of feed ratio and TSD on MAb titer at harvest day as well as one-way Anova study comparing the MAb titer at harvest (optimized process vs. baseline process in all runs)

Previous experiments performed with different daily fixed volume feed additions showed a correlation between feeding strategy and specific MAb productivity. It was observed that a significant decrease in the specific MAb productivity occurred if the feed ratio per the projection of a subset of process performance attributes was below a specific threshold (data not shown). A feed addition strategy based on the projected subset of process performance attributes was then developed. Based on previous screening study, feed ratio from 0.004 to 0.006 arbitrary units and and TSD from 0.30 to 0.40 arbitrary units were assessed. Custom DoE was performed with JMP SAS to study the interactions between both parameters. Number of interactions between the factors and the power of each factor were both fixed at 2. In total, 12 bioreactors were run. This Design of Experiment (DoE) was applied to the process development of a cell line 1 producing a monoclonal antibody and led to a 36% increase in the monoclonal antibody titer compared to control condition (Figure 1). The final feed ratio was based on (i) the improvement of MAb titer compared to the control condition, (ii) the scalability of the process (Culture start volume high enough to cover the impellers and low enough in order for Culture final volume to not exceed the maximum volume of the production bioreactor at large scale). TSD was fixed at 0.35 arbitrary units, so that a minimum dilution factor of 1:5 between the N-1 passage and the production bioreactor is achievable.

**Table 1:** MAb titers and Product Quality Attributes observed during the feed ranging study

	MAb titer (Normalized)	APG (Normalized)	Aggregate (Normalized)	Mannose 5 (Normalized)
Center point (n = 2)	1.00	1.00	1.00	1.00
+20% Feed (n = 2)	0.54	0.97	0.95	1.78
-20°% Feed (n = 2)	1.10	1.01	1.04	0.94

### Feeding strategy simplification, mode of feed addition, feeding ranging study

The design of the feeding strategy was simplified in order to facilitate the process transfer to large scale manufacture. Hence, based on the final feed ratio, the feed rates were fixed with a feed volume independent of the projected subset of process performance attributes. The pH of the feed is highly basic. In our 2L experiments, feed was added within less than 5 min, which generates pH excursions above 7.40. A strategy of slow bolus addition with a fixed minimum addition timeframe and with a fixed maximum flow rate was implemented, leading to minor pH-excursions during feeding with only minor CO_2 _flows necessary to keep the pH within the pH deadband (data not shown). The robustness of the process was assessed by performing an experiment with over- and underfeeding cultures. Underfeeding at 20% below target had no impact on process performance (MAb titer) while feeding 20% above target led to a lower MAb titer (Table 1). No impact of underfeeding or overfeeding at ± 20% of the feed target was observed on the Acidic Peak Group (APG) and aggregate levels. Feeding 20% above target led to an increase in Mannose 5 species.

## Conclusions

DoE enabled us to study the impact of the feed addition strategy and the impact of the TSD on the Mab titer and PQAs at harvest in a time efficient manner. The feeding strategy was simplified to become independent of the projected subset of process performance attributes and to be scalable to large scale manufacture. The mode of feed addition was optimized to minimize pH-excursions during feeding. Feed ranging studies showed that underfeeding at 20% below target had no impact on MAb titer and PQAs while feeding 20% above target led to a lower MAb titer and an increase in Mannose 5 species (glycan). Finally, a 36% increase in the MAb titer was achieved in the feed optimized conditions compared to control condition at harvest with a feed strategy designed to be robust and scalable.

